# Myeloid-derived suppressor cells in influenza virus-induced asthma exacerbation

**DOI:** 10.3389/fimmu.2024.1342497

**Published:** 2024-04-17

**Authors:** Chiel van Geffen, Tim Lange, Saeed Kolahian

**Affiliations:** ^1^ Institute of Laboratory Medicine, member of the German Center for Lung Research (DZL), The Universities of Giessen and Marburg Lung Center (UGMLC), Philipps University Marburg, Marburg, Germany; ^2^ Department of Experimental and Clinical Pharmacology and Pharmacogenomics, University of Tübingen, Tübingen, Germany; ^3^ Small Animal Imaging Core Facility, Center for Tumor Biology and Immunology (ZTI), Philipps University, Marburg, Germany

**Keywords:** myeloid-derived suppressor cells, asthma, influenza virus, asthma exacerbation, inflammation

## Abstract

Myeloid-derived suppressor cells (MDSCs) are a phenotypically heterogenous group of cells that potently suppress the immune response. A growing body of evidence supports the important role of MDSCs in a variety of lung diseases, such as asthma. However, the role of MDSCs in asthma exacerbation has so far not been investigated. Here, we studied the role of MDSCs in a murine model of influenza virus-induced asthma exacerbation. BALB/c mice were exposed to house dust mite (HDM) three times a week for a total of five weeks to induce a chronic asthmatic phenotype, which was exacerbated by additional exposure to the A/Hamburg/5/2009 hemagglutinin 1 neuraminidase 1 (H1N1) influenza virus. Induction of lung inflammatory features, production of T helper (Th) 1- and Th2- associated inflammatory cytokines in the lavage fluid and an increased airway hyper-responsiveness were observed, establishing the asthma exacerbation model. The number and activity of pulmonary M-MDSCs increased in exacerbated asthmatic mice compared to non-exacerbated asthmatic mice. Furthermore, depletion of MDSCs aggravated airway hyper-responsiveness in exacerbated asthmatic mice. These findings further denote the role of MDSCs in asthma and provide some of the first evidence supporting a potential important role of MDSCs in asthma exacerbation.

## 1 Introduction

Asthma exacerbations are a major global burden of disease, contributing to severe asthma progression, increased hospitalizations, and morbidity (1, 2). Total as well as asthma-related health care costs for patients with moderate to severe asthma are approximately doubled due to exacerbations alone (1). Onset of asthmatic exacerbation is typically accompanied with an exaggeration of existing inflammation, mucus hypersecretion and hyperresponsiveness of the airways (2). The most common triggers of asthma exacerbation are viral respiratory infections, with other common triggers being exposure to allergens, cigarette smoke and air pollutants (2). Respiratory infections that trigger exacerbations are most frequently induced by the human rhinovirus, but also by other viruses, such as influenza as was commonly observed during the 2009 influenza A virus (IAV) pandemic (2). Viral respiratory infections may lead to an enhanced sensitivity of the respiratory tract to allergens, and increases in local inflammatory responses, particularly of eosinophilic, neutrophilic and T cell-mediated responses, which in turn increase asthma exacerbation events (2–4). Despite significant efforts in asthma disease control with treatments such as corticosteroids, β-agonists, antileukotrienes, anticholinergics and monoclonal antibodies, asthma exacerbations still occur and remain difficult to treat (1, 2, 4). These complications persist primarily due to the complicated and heterogenous etiology of asthma and asthma exacerbation, and the remaining knowledge gap regarding its underlying mechanisms (2, 4).

Nevertheless, great strides have been made in unraveling the underpinning mechanisms, advancing our understanding beyond the initial hypothesized T helper (Th) 1/Th2 paradigm (3). Recent studies have shown the involvement of other immune cells, such as innate lymphoid cells (ILCs), monocytes and CD8^+^ T cells (3, 5). These findings further demonstrate the complexity of the disease, yet may result in novel therapeutics in the future, such as those targeting ILC2s, for which the monoclonal antibodies itepekimab (anti-IL33), astegolimab (anti-ST) and tezepelumab (anti-TSLP) are currently being studied (6). Myeloid-derived suppressor cells (MDSCs) are another immune cell population increasingly found to be involved in asthma (7). MDSCs are a heterogenous group of immature myeloid cells with potent immunosuppressive capacity (8). Since their discovery in cancer patients, they have been found to play a role in numerous diseases encompassing inflammation, infection, and autoimmunity (7–10). MDSCs are subdivided into polymorphonuclear (PMN-) and monocytic (M-) MDSCs, which are characterized by CD11b^+^Ly6G^+^Ly6C^int^ and CD11b^+^Ly6G-Ly6C^hi^ in mice, and CD11b^+^CD14^-^CD15^+^ and CD11b^+^CD14^+^HLA^-^DR^-/lo^CD15^-^ in humans, respectively (8, 11).

Previously, our group demonstrated the important role MDSCs play in different murine models of asthma (12, 13). These studies showed that the immunosuppressive capacity of MDSCs could be utilized to dampen lung inflammation in house dust mite (HDM)- and ovalbumin (OVA)-induced asthmatic mice by adoptive transfer of MDSCs (12, 13). Furthermore, we demonstrated that both the number and activity of MDSCs could be increased and that MDSCs were able to dampen airway inflammation in a HDM-induced murine model of asthma through a mechanism involving prostaglandin E2 (PGE2) subreceptor EP4 (13). Studying the potential role of MDSCs in asthma exacerbation could therefore be of paramount importance. However, the role MDSCs play in asthma exacerbation has, until now, not been investigated. In this study we aimed to investigate the role of MDSCs in IAV-induced asthma exacerbation, thereby further unraveling its underlying mechanisms and potentially expanding the possibilities on the development of novel therapeutic options in the future.

## 2 Results

### 2.1 Chronic exposure to HDM induced lung inflammatory features in a murine model of asthma exacerbation

To induce a severe and persistent allergic inflammatory response we adapted a chronic HDM model first described by Johnson et al. as described in 4.2 (14). Furthermore, to induce subsequent asthma exacerbation, HDM-induced asthmatic mice were subsequently exposed to IAV. To establish the IAV-induced model of asthma exacerbation, confirming the foundation of an underlying chronic asthma-like response was of paramount importance. The original model induced a severe and persistent eosinophilic inflammatory response, resulting in structural remodeling of the airways of HDM-exposed mice (14).

**Figure 1:**
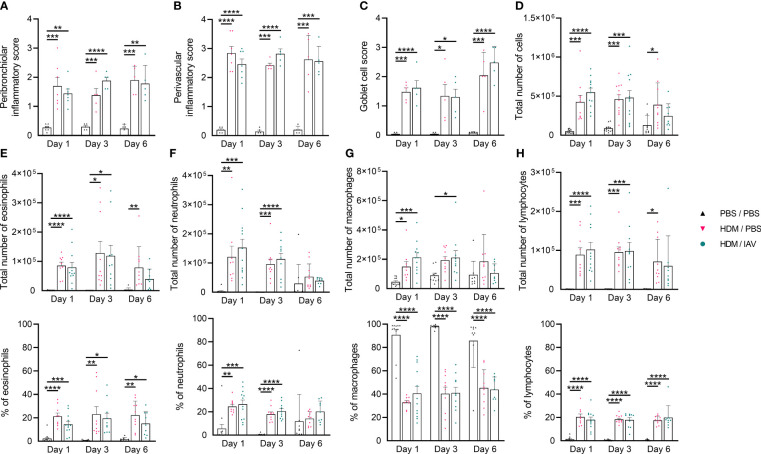
Chronic house dust mite (HDM) exposure induced lung inflammatory features. Mice were intranasally (i.n.) exposed three times a week for a total of five weeks to either phosphate buffered saline (PBS) or HDM. During the first exposure of the fifth week, mice were additionally i.n. exposed to either PBS or 2x10^5^ plaque-forming units (PFU) influenza A virus (IAV). One, three or six days after the final HDM exposure, the lung and bronchoalveolar lavage fluid (BALF) was collected and used to assess lung inflammatory features. **(A-C)** Peribronchiolar and perivascular inflammatory scores were assessed by H&E staining, **(C)** and the goblet cell score by periodic acid-Schiff (PAS) staining of lung sections. **(A-C)** Representative figures are presented in **Supplementary Figure 2**. **(D-H)** Differential counts were performed on collected BALF. **(D)** Total number of white blood cells (WBCs), **(E)** total number and % of eosinophils, **(F)** total number and % of neutrophils, **(G)** total number and % of macrophages and **(H)** the total number and % of lymphocytes are shown. **(A-C)** Data was obtained from 3-4 independent experiments, each performed with 1-2 mice per group, resulting in a total of 5-7 mice in each group. **(D-H)** Data was obtained from 5-6 independent experiments, each performed with 2 mice per group, resulting in a total of 10-12 mice in each group. **(A-H)** Symbols represent one individual mouse and bars indicate mean ± SEM. Statistical analysis was performed with one-way analysis of variance (ANOVA) followed by Tukey’s multiple comparisons test with the following p values: *p<0.05, **p<0.01, ***p<0.001, ****p<0.0001.

**Figure 2:**
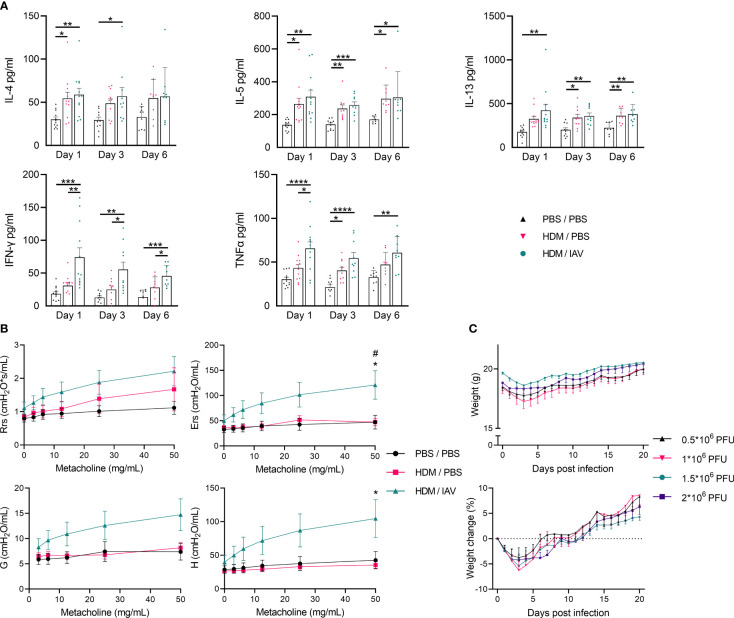
Model of asthma exacerbation promoted T helper (Th) 1/Th2 cytokine production, increased airway hyperresponsiveness and induced weight loss. Mice were i.n. exposed three times a week for a total of five weeks to either phosphate buffered saline (PBS) or house dust mite (HDM). During the first exposure of the fifth week, mice were additionally intranasally (i.n.) exposed to either PBS or 2x10^5^ plaque-forming units (PFU) influenza virus A (IAV). One, three or six days after the final HDM exposure, bronchoalveolar lavage fluid (BALF) was collected. **(A)** The Th2 cytokines IL-4, IL-5, and IL-13 as well as the Th1 cytokines interferon-gamma (IFN-γ), and tumor necrosis factor-alpha (TNF-α) were analyzed in the BALF supernatant *via* enzyme-linked immunosorbent assays (ELISAs). Lung function was measured prior to BALF collection *in vivo* using FlexiVent. Anesthetized mice were challenged with increasing doses of aerosolized methacholine (0; 3.125; 6.25; 12.5; 25 and 50 mg/ml). **(B)** Airway resistance (Rrs), airway elastance (Ers), tissue damping (G), and tissue elastance (H) were assessed. **(C)** Furthermore, mice that were similarly i.n. exposed three times a week for a total of five weeks to HDM, received higher i.n. dosages of IAV (0.5*10^6^; 1*10^6^; 1.5*10^6^; 2*10^6^ PFU) during the fifth week instead. **(C)** The weight of these mice was monitored over 21 days post infection and recorded weight as well as the relative weight change post infection are presented. **(A)** Data was obtained from 5-6 independent experiments, each performed with 2 mice per group, resulting in a total of 10-12 mice in each group. **(A)** Symbols represent one individual mouse and bars indicate mean ± SEM. **(B)** Data was obtained from 3 independent experiments, each performed with 2 mice per group, resulting in a total of 6 mice in each group. **(C)** Data was obtained from 4 independent experiments, each performed with 5 mice per group, resulting in a total of 20 mice in each group. **(B, C)** Means ± SEM of the pooled data are shown. Statistical analysis was performed with one-way analysis of variance (ANOVA) followed by Tukey’s multiple comparisons test with the following p values: **(A)** *p<0.05, **p<0.01, ***p<0.001, ****p<0.0001, **(B)** #p<0.05: comparing PBS/PBS vs HDM/IAV, *p<0.05: comparing HDM/PBS vs HDM/IAV.

To assess whether the adapted model achieved similar results, features of the inflammatory response were assessed on day 1, day 3, and day 6 after the last HDM exposure (**Figures 1**, **2**, **Supplementary Figure 1A**). First, we analyzed histopathological inflammatory features of the lung (**Figures 1A-C**, **Supplementary Figure 2**). HDM exposure resulted in an inflammatory response of the airways, as indicated by the significantly increased peribronchiolar and perivascular inflammatory and goblet cell scores of both asthmatic (HDM/phosphate buffered saline (PBS)) and exacerbated asthmatic (HDM/IAV) mice compared to controls (PBS/PBS) (**Figures 1A-C**). The increase in inflammatory scores remained relatively constant between day 1 to day 6, with a slight increase in goblet cell score at day 6, compared to earlier time points. No statistically significant changes of inflammatory scores were observed between the exacerbated and non-exacerbated asthmatic mice.

Next, differential counts of white blood cells (WBCs) within the sediment of the bronchoalveolar lavage fluid (BALF) were performed and analyzed (**Figures 1D-H**). The total number of WBCs, eosinophils, neutrophils, macrophages, and lymphocytes significantly increased in both asthmatic and exacerbated asthmatic mice compared to PBS controls (**Figures 1D-H**). While the total number of neutrophils seemed to peak on day 1, and the total number of eosinophils peaked on day 3, all cells showed reduced numbers on day 6. Again, no statistically significant differences were observed between the exacerbated and non-exacerbated asthmatic mice.

### 2.2 Chronic exposure to HDM increased the production of Th2-associated inflammatory cytokines

To further determine the impact of chronic HDM exposure and subsequent IAV exposure in our adapted mice model, we performed quantitative analysis of the main Th1- and Th2-associated cytokines found within the BALF with enzyme-linked immunosorbent assays (ELISAs) (**Figure 2A**). The concentration of the Th2-associated cytokines IL-4, IL-5 and IL-13 were found to be increased in asthmatic and exacerbated asthmatic mice, with no statistically significant difference observed between these two groups, besides a slight upwards trend in exacerbated asthmatic mice (**Figure 2A**). Although the increase in IL-4 and IL-13 did not consistently show significant increases in all end point day data, perhaps explained by the variation within some groups after combining data from 5-6 independent experiments, the upward trend remains apparent.

### 2.3 Subsequent IAV exposure also increased the production of Th1-associated inflammatory cytokines

The concentration of the Th1-associated cytokines interferon-gamma (IFN-γ) and tumor necrosis factor-alpha (TNF-α) is significantly increased in exacerbated asthmatic mice compared to PBS controls (**Figure 2A**). IFN-γ concentrations are also significantly increased in exacerbated compared to non-exacerbated asthmatic mice during all three different endpoint days, with TNF-α concentrations only being significantly higher on day 1 between non-exacerbated and exacerbated asthmatic mice. IFN-γ concentrations in the BALF of exacerbated asthmatic mice showed a slight downward trend over time from day 1 to day 6 (**Figure 2A**). The induction of lung inflammatory features and increased production of Th2 cytokines in the BALF following HDM exposure both point towards an asthma-like response and similar features were observed in previous HDM-induced murine models of asthma (12, 13). The Th1 inflammatory cytokine response in the BALF, induced after additional exposure to IAV, suggests the asthma-like response may be aggravated, providing the first clues towards establishing the asthma exacerbation model.

### 2.4 IAV aggravated airway hyper-responsiveness in HDM-induced asthmatic mice.

To assess the impact of subsequent IAV exposure on the lung function of asthmatic mice we assessed airway hyperresponsiveness *in vivo* by measuring lung function during exposure to incremental doses of nebulized methacholine as described in 4.5. Airway resistance (Rrs), airway elastance (Ers), tissue damping (G), and tissue elastance (H) showed apparent upward trends in exacerbated asthmatic mice compared to both PBS controls as well as non-exacerbated asthmatic mice, although only reaching statistical significancy in airway elastance compared to both PBS control and non-exacerbated asthmatic groups as well as in tissue elastance compared to PBS control, at the highest dose of methacholine (**Figure 2B**). The level of airway resistance of non-exacerbated asthmatic mice seemed to fit in an intermediate stage between PBS control and exacerbated asthmatic mice (**Figure 2B**). Strikingly, airway elastance, tissue damping and tissue elastance appeared relatively unaffected in non-exacerbated asthmatic mice (**Figure 2B**). While HDM exposure appeared to moderately impair lung function by increasing airway resistance, subsequent IAV exposure aggravated all measured lung function parameters, suggesting severely impaired lung functions. Additionally, the bodyweight of HDM-induced asthmatic mice that were exposed to increased doses of IAV were found to drop abruptly during the first 3 days following infection, after which it recovers back to the starting weights in the subsequent week (**Figure 2C**, **Supplementary Figure 1B**). No significant changes were observed in lung inflammatory features, BALF inflammatory cytokines, nor in the number and activity of pulmonary MDSCs in asthmatic mice exposed to increased doses of IAV 21 days post infection (data not shown). These findings further confirm the acute response to IAV infection also observed in previous studies, resulting in acute exacerbation of the asthma-like response which, based on the changes in body weight appeared to last several days, after which a recovery phase set in (15, 16).

### 2.5 IAV-induced exacerbation of asthmatic mice increased the number of pulmonary M-MDSCs

**Figure 3:**
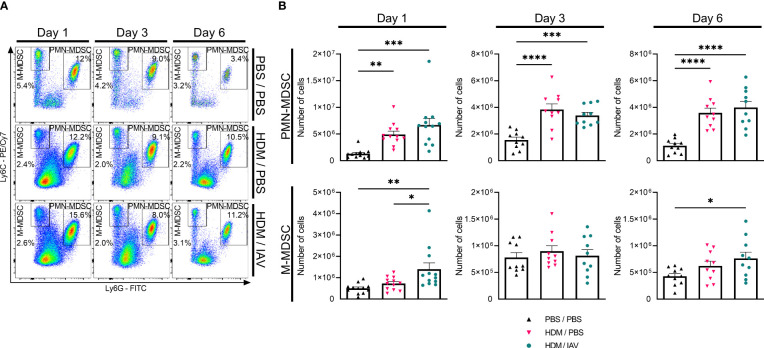
Exacerbation of asthmatic mice increased the number of lung monocytic myeloid-derived suppressor cells (M-MDSCs) on day 1 and day 6. Mice were intranasally (i.n.) exposed three times a week for a total of five weeks to either phosphate buffered saline (PBS) or house dust mite (HDM). During the first exposure of the fifth week, mice were additionally i.n. exposed to either PBS or 2x10^5^ plaque-forming units (PFU) influenza A virus (IAV). One, three or six days after the final HDM exposure, the lungs were collected. **(A, B)** Polymorphonuclear (PMN)- and M-MDSCs were subtyped using flow cytometry. **(A)** Representative figures of the MDSC gating process of CD11b^+^ cells from the lungs, based on the Ly6C and Ly6G markers. **(B)** Quantitative results of the number of PMN- and M-MDSCs in the lungs. The percentages of MDSCs were used together with the total cell counts of the lung to calculate the total number of MDSCs in the lung. Data was obtained from 5-6 independent experiments, each performed with 2 mice per group, resulting in a total of 10-12 mice in each group. Symbols represent one individual mouse and bars indicate mean ± SEM. Statistical analysis was performed with one-way analysis of variance (ANOVA) followed by Tukey’s multiple comparisons test with the following p values: *p<0.05, **p<0.01, ***p<0.001, ****p<0.0001.

After establishing a body of evidence that seemed to sufficiently confirm and establish our murine model of asthma exacerbation, we set out to study the role of MDSCs. To quantify the number of MDSCs we subtyped both PMN- and M-MDSCs in the single cell suspensions of collected lungs (**Figure 3**, **Supplementary Figure 1A**). The number of PMN-MDSCs in the lungs of asthmatic mice was significantly increased on all three endpoint days compared to PBS control mice, with no statistically significant difference between exacerbated and non-exacerbated asthmatic mice (**Figure 3**). In contrast, the number of M-MDSCs was found to be significantly increased in the lungs of exacerbated asthmatic mice compared to both PBS control and non-exacerbated asthmatic mice on day 1 as well as compared to PBS control on day 6. Strikingly, the rise in the number of M-MDSC was found to be abolished on day 3.

### 2.6 IAV-induced exacerbation of asthmatic mice further augmented the inhibitory effect of lung MDSCs on T cell proliferation

**Figure 4:**
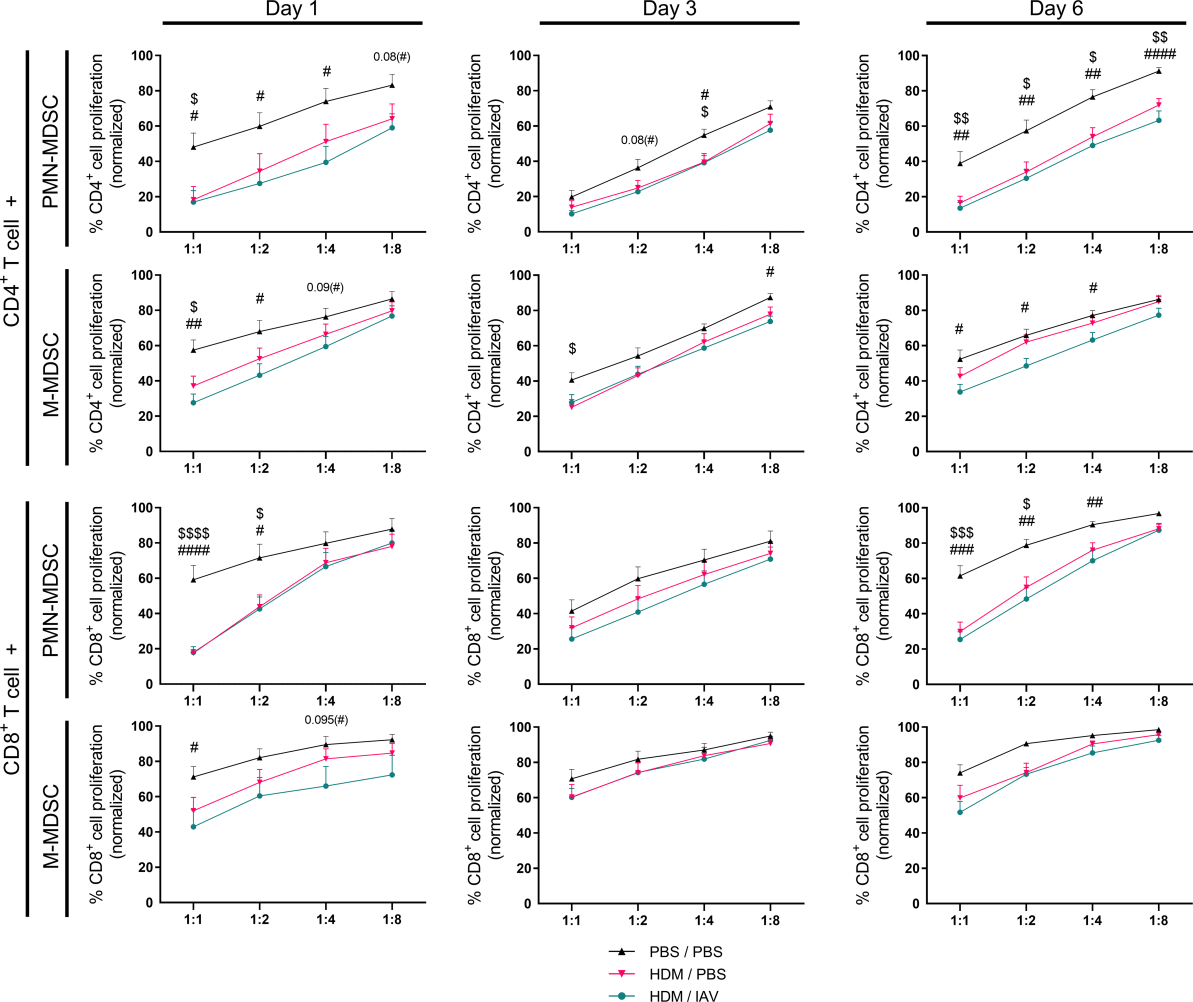
The immunosuppressive activity of lung myeloid-derived suppressor cells (MDSCs) increased in asthmatic mice, and slightly increased in exacerbated asthmatic mice overall. Mice were intranasally (i.n.) exposed three times a week for a total of five weeks to either phosphate buffered saline (PBS) or house dust mite (HDM). During the first exposure of the fifth week, mice were additionally i.n. exposed to either PBS or 2x10^5^ plaque-forming units (PFU) influenza A virus (IAV). One, three or six days after the final HDM exposure, the lungs were collected. Both polymorphonuclear (PMN)- and monocytic (M)- myeloid-derived suppressor cells (MDSCs) were isolated from the lungs by magnetic-activated cell sorting (MACS) separation. Isolated MDSCs were then co-cultured with CD3/CD28 biotin-beads-stimulated and carboxyfluorescein succinimidyl ester (CFSE)-stained CD4^+^ T cells or with CD8^+^ T cells isolated from spleens of naïve donor mice at different ratios (MDSC:T cell ratios of 1:1, 1:2, 1:4 and 1:8). Proliferation of T cells was assessed after three days of co-culture by measuring CFSE dilution. Proliferation of T cells was normalized to the positive control (CD3/CD28-stimulated T cells only, set to 100% proliferation). Representative figures are presented in **Supplementary Figure 3**. Data was obtained from 5-6 independent experiments, each performed with 2 mice per group, resulting in a total of 10-12 mice in each group (N=12 for each group of day 1, N=10 for each group of day 3 and day 6). Means ± SEM of the pooled data are shown. Statistical analysis was performed with one-way analysis of variance (ANOVA) followed by Tukey’s multiple comparisons test with the following p values: $p<0.05, $ $p<0.01, $ $ $p<0.001, $ $ $ $p<0.0001 comparing PBS/PBS vs HDM/PBS; #p<0.05, ##p<0.01, ###p<0.001, ####p<0.0001 comparing PBS/PBS vs HDM/IAV.

We then studied the immunosuppressive capacity of MDSCs isolated from the lungs. Immunosuppressive activity was assessed by measuring the impact of both PMN- and M-MDSCs on the proliferation of naïve CD4^+^ or CD8^+^ T cells (**Figure 4**, **Supplementary Figure 3**). Immunosuppressive activity of both PMN- and M-MDSCs isolated from the lungs of asthmatic mice was found to be increased on both CD4^+^ and CD8^+^ T cells, as indicated by the inhibited proliferation of T cells that were co-cultured with MDSCs, on all three end point days compared to PBS controls, albeit not always reaching statistical significancy (**Figure 4**). MDSCs inhibited the proliferation of both CD4^+^ and CD8^+^ T cells to a similar extent, indicating there is no preferential impact of MDSCs’ immune suppression in this model. The immunosuppressive activity in the non-exacerbated and exacerbated asthmatic mice seemed to generally overlap, yet slight trends of increased immunosuppressive activity were observed in the exacerbated asthmatic mice group, especially M-MDSCs activity on day 1 on both CD4^+^ and CD8^+^ T cells and on day 6 on CD4^+^ T cells. Interestingly, the increased activity observed on day 1 and day 6 appears to correlate with the days we observed an increased numbers of MDSCs, particularly M-MDSCs, in the lung (**Figures 3**, **4**).

### 2.7 Depletion of MDSCs further aggravated airway hyper-responsiveness in a model of asthma exacerbation.

**Figure 5:**
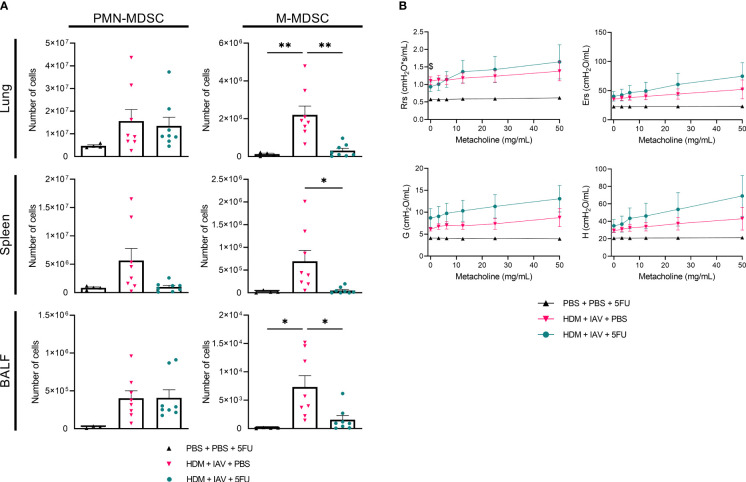
5-fluorouracil (5FU) depleted monocytic myeloid-derived suppressor cells (M-MDSCs), as well as splenic polymorphonuclear (PMN)-MDSCs and further aggravated lung function in exacerbated asthmatic mice. Mice were intranasally (i.n.) exposed three times a week for a total of five weeks to either phosphate buffered saline (PBS) or house dust mite (HDM). Starting one day before the first HDM challenge, 5FU was administered intraperitoneally (i.p.) weekly to deplete MDSCs. During the first exposure of the fifth week, mice were additionally i.n. exposed to either PBS or 2x10^5^ plaque-forming units (PFU) influenza A virus (IAV). One day after the final HDM exposure, the lungs, spleens and bronchoalveolar lavage fluids (BALF) were collected. **(A)** PMN- and M-MDSCs were subtyped using flow cytometry. Representative figures of the MDSC gating process are presented in **Supplementary Figures 4**, **5**. **(A)** Quantitative results of the number of PMN- and M-MDSCs in the lungs. **(B)** Prior to organ collection the lung function was measured *in vivo* using FlexiVent. Anesthetized mice were challenged with increasing doses of aerosolized methacholine (0; 3.125; 6.25; 12.5; 25 and 50 mg/ml). **(B)** Airway resistance (Rrs), airway elastance (Ers), tissue damping (G), and tissue elastance (H) were assessed. **(A, B)** Data was obtained from 4 independent experiments, each performed with 1 mouse in the PBS/PBS/5FU group and 2 mice in the remaining groups, resulting in a total of 4 mice in the PBS/PBS/5FU group and 8 mice in each of the remaining groups. **(A)** Symbols represent one individual mouse and bars indicate mean ± SEM. **(B)** Means ± SEM of the pooled data are shown. Statistical analysis was performed with one-way analysis of variance (ANOVA) followed by Tukey’s multiple comparisons test with the following p values: *p<0.05, **p<0.01.

To gain further insight into the role MDSCs play in asthma exacerbation, we attempted to deplete MDSCs during the model of asthma exacerbation with adding recurring injections of 5-fluorouracil (5FU) as described in 4.4 (**Supplementary Figure 1C**). To assess whether and to what extent MDSCs were depleted, we subtyped both PMN- and M-MDSCs and quantified their numbers in lungs, spleens and BALF (**Figure 5A**, **Supplementary Figures 4**, **5**). 5FU injections successfully depleted M-MDSCs, yet PMN-MDSCs were only found to be depleted in the spleens. Indeed, the number of PMN-MDSCs remained at similar levels to exacerbated asthmatic mice that did not receive 5FU in both the lungs and BALFs. Then, the impact of (partial) MDSC depletion on lung function in a murine model of asthma exacerbation was assessed. Airway hyper-responsiveness was again measured *in vivo* by measuring lung function during exposure to incremental doses of nebulized methacholine. Here, the airway resistance, airway elastance, tissue damping, and tissue elastance all show an upward trend in exacerbated asthmatic mice compared to controls (**Figure 5B**). Notably, 5FU treatment alone had no effect on lung size and function and induced no obvious signs of airway toxicity (data not shown). These findings demonstrate that the depletion of MDSCs further aggravated airway hyper-responsiveness as observed by the apparent trend of increased respiratory parameters compared to non-depleted exacerbated asthmatic mice.

## 3 Discussion

An increasing body of evidence suggests that MDSCs play a crucial role in lung diseases such as lung cancer, chronic obstructive pulmonary disorder (COPD), tuberculosis, pulmonary hypertension, cystic fibrosis, pulmonary infection, and asthma (7, 10). Our previous findings further underlined the important role of MDSCs in murine models of asthma (12, 13). However, the possible role MDSCs play in asthma exacerbation had previously not been studied yet. Here, for the first time, the role of MDSCs in a murine model of asthma exacerbation was examined (**Supplementary Figure 6**). We found that the number as well as the immunosuppressive activity of pulmonary MDSCs, particularly of M-MDSCs, increased following IAV-induced exacerbation of HDM-induced asthma. Furthermore, MDSC depletion further aggravated airway hyperresponsiveness induced by the asthma exacerbation model. Collectively, these findings show that MDSCs, particularly M-MDSCs, play an important role in IAV-induced asthma exacerbation by inhibiting T cell proliferation and thereby contributing to dampened respiratory symptoms. Besides the impact of MDSCs on T cell proliferation it would be very interesting to also assess the impact on T cell function in future research to expand on other aspects of potential suppressive characteristics.

A rise in the number as well as in the immunosuppressive activity of MDSCs has previously been observed by our group in experiments including murine OVA- and HDM-induced asthma models, and similar observations have been made in a multitude of other asthma-related studies as well (7, 12, 13, 17). In line with those findings, we found that long-term HDM exposure, as was used in the models in this study, increased both the number as well as the immunosuppressive activity of PMN- and M-MDSCs in the lung. Akin to the findings in murine asthma models, pulmonary viral infections, triggered by viruses such as influenza, have been reported to induce expansion of immunosuppressive pulmonary MDSCs in mice (10, 18). In the asthma exacerbation model used in this study, long-term exposure to HDM was combined with a one-time exposure to IAV. We found that IAV-induced exacerbation of the HDM-induced asthma-like response further increased the number and activity of pulmonary MDSCs compared to non-exacerbated asthmatic mice. These findings seem to suggest that there is an additive effect of the IAV-induced infection resulting in an enhanced induction of MDSC expansion and activity initially triggered by the HDM exposure preceding it. The IAV-induced asthma exacerbation model was further established with findings such as the IAV-induced increases in the production of Th1-associated inflammatory cytokines as well as the IAV-induced aggravation of airway hyper-responsiveness in HDM-induced asthmatic mice. Furthermore, a previous study that assessed immune cell populations in the BALF of atopic asthmatics showed that the number of MDSCs more than doubled following segmental allergen challenge (19). Despite not being directly comparable to our experimental model, these observations in human asthma seem to further corroborate our findings of increased MDSCs in the lung and BALF following allergen challenge. Taken together, these findings suggest that the additive increases in the number and activity of pulmonary MDSCs following IAV infection are associated with the IAV-induced exacerbation of the HDM-induced asthma-like response.

The kinetics and severity of IAV infections can vary significantly depending on the strain of the virus as well as the host (16, 20, 21). For example, the pandemic IAV strain A/Hamburg/5/2009 used in this study was found to display higher pathogenicity in C57BL/6J mice compared to BALB/c mice, possibly due to the lack of importin-α7 (an isoform of an adaptor protein that binds the nuclear localization signal, allowing transport of proteins from the cytoplasm to the nucleus) in the BALB/c mice strain (21, 22). The relatively low pathogenicity of this IAV strain, especially in BALB/c mice, was confirmed by the high survival rate of mice in experiments with increasing doses of IAV used in the asthma exacerbation model (data not shown). However, the dose of 2x10^5^ plaque-forming units (PFU) of this IAV strain was previously found to induce airway inflammation and weight loss in BALB/c mice (15, 16). Our findings suggest similar pathogenicity within our asthma exacerbation model using BALB/c mice, which contributed to the observed IAV-induced exacerbation of the HDM-induced asthma model. The choice for BALB/c mice instead of the more IAV-susceptible C57BL/6J mice for the asthma exacerbation model was also based on the fact that BALB/c mice are the most commonly used mouse strains in asthma models as they have been found to develop a Th2-biased immunological response, which is generally considered to be more representative to human asthma (23). Furthermore, while IAV is a common trigger, it would perhaps also be interesting to replace it within the model with other triggers, especially rhinoviruses, since those are the most common viral respiratory triggers of asthma exacerbation (2). We are currently stabilizing other murine models of virus-induced asthma exacerbation, where the pandemic Hamburg/5/2009 IAV strain is replaced by a Puerto Rico/8/1934 IAV virus (PR8).

Previous research has shown that the recruitment and activation of MDSCs is mediated by a multitude of factors, including cytokines such as IL-4 and IL-13, and chemokines, including CCL2 and CXCL2 (8, 24). However, the molecular mechanisms by which MDSCs regulate airway inflammation in asthma exacerbation remained poorly defined. To further assess the kinetics of the MDSC response in the asthma exacerbation model we looked at three different endpoints for our experiments, including day 1, 3 and 6 following the last HDM exposure. As previously noted, the fluctuations in the number of MDSCs relative to MDSC activity, particularly of M-MDSCs, were found to correlate on the different endpoint days. These findings may hint at complex temporal patterns of the MDSC response in relation to the model of asthma exacerbation. While the increase in MDSC number is likely a result of enhanced recruitment of MDSCs to the lung, the changes in MDSC activity may be directly related to changes in the micro-environment of the lungs, such as those induced by rapid alterations in cytokine production, following IAV infection (7, 8, 10, 18). The observed increase of IL-4 and IL-13 in the BALF of asthmatic mice suggests a local increase in MDSC activity, as both cytokines have been shown to induce MDSC activation (8). Furthermore, while we showed that respiratory symptoms worsened following MDSC depletion, the normal MDSC response is unable to prevent the mice in our model from reaching asthmatic stages, suggesting that the overall mechanism is closely linked to model-induced inflammatory cytokine production. Further studies are needed to unravel the exact mechanisms behind the impact of IAV-induced exacerbation of asthma on the inflammatory processes resulting in MDSC recruitment, transitioning and re-emergence in MDSC number and activity. However, since a convincing number of results pointed towards day 1 being closest to the peak of asthma exacerbation within our model, while simultaneously showing the highest number of discrepancies between non-exacerbated and exacerbated asthmatic mice, we decided to focus on day 1 for the depletion experiments.

Since the identification of MDSCs and the implications of their immunosuppressive activity within the context of the tumor environment, a considerable effort has been made to find ways to dampen their number and activity (8, 9, 11, 25–28). Chemotherapeutic drugs, such as gemcitabine and 5FU, functioning as mono- or combination therapies in the treatment of certain tumors, such as breast and pancreatic cancer, were found to significantly reduce MDSC numbers (25–28). While chemotherapeutic agents do not specifically target MDSCs, they were found to deplete MDSCs with relative specificity in different murine models, with 5FU showing stronger efficacy overall (26, 28, 29). Repeating doses of 50 mg/kg 5FU were shown to effectively deplete MDSCs in mice while not significantly affecting T cells, B cells, natural killer cells (NKs) and dendritic cells (DCs) (26, 29). Although adverse toxicity, including lung toxicity, has been reported in clinically relevant dosages of 5FU (30), we did not observe significant adverse effects of 5FU on the lungs in our depletion model (data not shown). While 5FU successfully depleted both MDSC subsets in the spleen, only M-MDSCs were found to be depleted in the lungs and BALF, suggesting the pulmonary PMN-MDSCs subset evades the 5FU-induced depletion at this dosage at least partially. Higher or more frequent 5FU dosages may be necessary to achieve complete depletion of the pulmonary PMN-MDSC subset, yet may result in adverse effects, such as pulmonary toxicity. Monoclonal antibodies targeting murine markers of MDSCs, such as the α-Gr1 and α-Ly6C antibodies, could function as effective alternatives to chemotherapeutic agents, yet are categorically limited to depleting either PMN- or M-MDSC subsets, respectively (31).

Great strides have been made in understanding the underlying mechanisms in asthma and asthma exacerbation, leading to the identification of new immune components, such as ILC2s, monocytes, CD8^+^ T cells and MDSCs (3, 5). There is an emerging body of evidence supporting the application of the immunosuppressive potential of MDSCs to dampen inflammation, such as in asthma (13, 17, 32, 33). This study further underlines the important role of MDSCs in asthma as well as providing novel insights into their role in asthma exacerbation. MDSC depletion resulted in amplifying the IAV-induced exacerbation of asthma, hinting at a crucial and beneficial role of MDSCs to dampen the induced inflammatory response. Therefore, deciphering the exact mechanisms underlying the immunomodulatory role of MDSCs may further unlock their immunosuppressive potential in asthma exacerbation, as well as in other inflammatory diseases, and may lead the way to potential novel therapeutic strategies targeting MDSCs in the near future (10, 32, 33).

## 4 Material and methods

### 4.1 Mice

Experiments using animals were approved by the Regierungspräsidium Tübingen, Germany and by the Regierungspräsidium Giessen, Germany (protocol PH 01/G21-2021, G21-2021 Transfer) and were carried out according to the Directive 2010/63/EU and German legislation. Six- to eight-week-old female BALB/c mice were purchased from Charles River Laboratories (Sulzfeld, Germany). The mice were housed in individually ventilated cages and provided with food and water ad libitum. A 12-hour light-dark cycle within a controlled specific pathogen-free environment was maintained for all animals. After a brief acclimatization period mice were randomly assigned to the different experimental groups.

### 4.2 Murine model of HDM- and subsequent IAV-induced asthma exacerbation

We adapted a chronic HDM model first described by Johnson et al. (14). In our adapted model the mice were intranasally (i.n.) treated three times a week for five consecutive weeks with HDM extract (Greer Laboratories, Lenoir, NC, USA) or PBS (**Supplementary Figure 1A**). Lyophilized HDM extracts were resuspended in PBS so that each i.n. application contained 25 µg (protein) in a volume of 40 µl.

The swine-origin pandemic influenza virus A/Hamburg/5/2009 (hemagglutinin 1 neuraminidase 1 [H1N1]; further referred to as IAV) was propagated using Madin-Darby canine kidney (MDCK) cells (16). Infectivity was quantified via plaque assay using MDCK cells and expressed as PFU per milliliter. IAV aliquots were stored at -80°C until use. Subsequent exacerbation of the HDM-induced allergic inflammatory response was triggered on the first day of the final week of HDM challenges by the additional i.n. application of 2x10^5^ PFU of the virus (or PBS for controls) diluted in PBS to a volume of 40 µl. Mice were sacrificed for endpoints one, three and six days after the last HDM challenge, which was equivalent to three, six and nine days post IAV infection, respectively (**Supplementary Figure 1A**). All i.n. applications were performed under light anesthesia with isoflurane.

### 4.3 Increased IAV exposure

Experiments were performed where the dosage of IAV in the asthma exacerbation model as described above was increased (**Supplementary Figure 1B**). The dosage of i.n. IAV application was increased from 2x10^5^ to 5x10^5^, 10x10^5^, 15x10^5^ or 20x10^5^ PFU, all diluted in PBS to an end volume of 40 µl/dose. Surviving mice were sacrificed 21 days post infection for further analysis. During all experiments mice were closely monitored according to guidelines, and, if necessary, killed prematurely to minimize animal suffering.

### 4.4 MDSC depletion

MDSCs were depleted by intraperitoneal (i.p.) injections of 5FU (50 mg/kg, Sigma-Aldrich). Mice were injected with 5FU one day before the first HDM treatment of every week of the IAV-induced asthma exacerbation model, for a total of 5 doses (**Supplementary Figure 1C**). 5FU was dissolved in PBS and injected at a volume of 200 µl, with control mice receiving PBS only.

### 4.5 Airway responsiveness

Airway responsiveness was assessed at the indicated time points by methacholine-induced airway responses in anesthetized mice using the FlexiVent FX platform (SCIREQ, Montreal, Canada). Mice were anesthetized by i.p. injections of ketamine (120 mg/kg, Serumwerk Bernburg Tiergesundheit, Bernburg, Germany) and medetomidine (1 mg/kg, Orion Pharma, Zug, Switzerland). Tracheotomy was subsequently performed, and a cannula was inserted to connect the mice to the FlexiVent FX platform, after which the mice were mechanically ventilated at 150 breaths/min, with a tidal volume of 10 ml/kg and a positive end expiratory pressure of 3 cmH2O. To prevent spontaneous respirations during the measurements, an i.p. injection of 0.8 mg/kg pancuronium (Inresa Arzeimittel, Freiburg, Germany) was administered. The degree of airway responsiveness was assessed by exposure to incremental concentrations of aerosolized methacholine (Merck, Germany). During this procedure, the airway resistance, airway elastance, tissue elasticity and tissue damping were measured using forced oscillation techniques.

### 4.6 Bronchoalveolar lavage

At the end of the model mice were sacrificed for further analysis at the indicated time points. To collect the BALF, lungs were flushed three times with 0.8 ml PBS, supplemented with 0.5M ethylenediaminetetraacetic acid (EDTA). Supernatants were stored until further analysis and the lavage pellets were prepared for differential counts on Cytospins. Cytospin preparations were stained according to Pappenheim, with Giemsa (Carl Roth, Karlsruhe) and May-Grünwald (Merck) stains. Differential counts of eosinophils, neutrophils, macrophages, and lymphocytes were performed on a total of 400 cells per sample.

### 4.7 ELISA

The levels of IL-4, IL-5, IL-13, IFN-γ and TNF-α were measured in the supernatants of the collected BALF using ELISA kits according to manufacturer’s protocol (R&D System, Minneapolis, USA).

### 4.8 Single cell preparation

Spleens, lungs and draining lymph nodes were collected and single cell suspensions were prepared. The lung was digested using 200 U/ml collagenase type IV, 25 U/ml DNAse I and 2.5 mM MgCl2 dissolved in Hanks’ Balanced Salt Solution (all from Thermo Fisher) at 37°C for 45 minutes with continuous agitation, after being thoroughly minced. Digested lung tissue, spleens and lymph nodes were filtered through 70 and then 40 µm strainers. Red blood cells were then lysed, if necessary, using ammonium-chloride-potassium (ACK) lysing buffer.

### 4.9 Flow cytometry

Obtained single cell suspensions were stained with different panels of fluorescent labeled antibodies and analyzed by flow cytometry using the BD FACSCanto II (BD Biosciences) and the CytoFLEX (Beckman Coulter, Brea, California, USA). MDSCs were characterized using CD11b-APC (M1/70), Ly6G-FITC (1A8) and Ly6C-PE/Cy7 (HK1.4) antibodies (all from BioLegend, **Figure 3**, **Supplementary Figures 4**, **5**). Total number of MDSCs were calculated by combining the MDSC percentages together with the total cell counts of the prepared single cell suspensions. Flow cytometry data was analyzed using FlowJo software (FlowJo LLC, Ashland, OR, USA).

### 4.10 Measuring MDSC suppressive activity

Both PMN-MDSCs (by positive selection of Ly6G) and M-MDSCs (by positive selection of Gr1 of Ly6G depleted cell suspension) were isolated from the obtained single cell suspensions of the lung and spleen using the magnetic-activated cell sorting (MACS) MDSC isolation kit (Miltenyi Biotec, Bergisch Gladbach, Germany) according to manufacturer’s instructions. CD4^+^ and CD8^+^ T cells were isolated from the spleens of naïve BALB/c mice using MACS T cell isolation kits (Miltenyi Biotec) by negative selection according to manufacturer’s protocol. Isolated T cells were then stained with 1 µmol/L carboxyfluorescein succinimidyl ester (CFSE; BioLegend). CFSE-labeled CD4^+^ and CD8^+^ T cells were seeded in U-shaped 96-well plates at 1x10^5^ cells per well and co-cultured with isolated PMN- or M-MDSCs at different ratios. Culture media consisted of RPMI 1640 supplemented with 10% FBS, 2 mM L-glutamine, 1% penicillin/streptomycin, 50 µM 2-mercaptoethanol together with 100 U/ml IL-2 (BioLegend) and anti-biotin MACSiBead particles loaded with CD3ϵ- and CD28-biotin (Miltenyi Biotec) with a bead-to-cell ratio of 1. After three days of co-culture the proliferation of CFSE-labeled CD4^+^ and CD8^+^ T cells was analyzed by flow cytometry (**Supplementary Figure 3**).

### 4.11 Histopathology

The left diaphragmatic lobe of the lungs was immediately fixated with 4% phosphate-buffered formalin (Carl Roth) before being dehydrated and embedded in paraffin. Lung sections were stained with hematoxylin and eosin to assess perivascular and peribronchial inflammation and with periodic acid-Schiff (PAS) stain (Carl Roth) to quantify mucus-containing goblet cells in bronchi. Severity of perivascular and peribronchial inflammation was evaluated by scoring the number of inflammatory cells formed around the vessels and airways from grade 0 to grade 4 as follows: 0, no inflammation; 1, scattered inflammatory cells; 2, one ring of inflammatory cells; 3, two- to four layers of inflammatory cells; 4, more than four layers of inflammatory cells (16). PAS-positive goblet cells were semi-quantified by scoring from grade 0 to grade 4 as follows: 0, <5% PAS-positive cells; 1, 5-25%; 2, 25-50%; 3, 50-75%; 4, >75%.

### 4.12 Statistics

All statistical analyses were performed in GraphPad Prism version 9 (San Diego, USA). Data sets were analyzed by one-way analysis of variance (ANOVA) followed by Tukey’s multiple comparisons test unless specified otherwise. Statistical significance was determined with a p value of 0.05 or lower. Results are presented as mean ± SEM unless specified otherwise. A p value of less than 0.05 was considered statistically significant.

## Data availability statement

The raw data supporting the conclusions of this article will be made available by the authors, without undue reservation.

## Ethics statement

The animal study was approved by the Regierungspräsidium Tübingen, Germany and by the Regierungspräsidium Giessen, Germany (protocol PH 01/G21-2021, G21-2021 Transfer). The study was conducted in accordance with the local legislation and institutional requirements.

## Author contributions

CvG: Formal Analysis, Methodology, Visualization, Writing – original draft, Writing – review & editing, Conceptualization, Investigation, Project administration. TL: Investigation, Writing – review & editing. SK: Conceptualization, Data curation, Formal Analysis, Funding acquisition, Investigation, Methodology, Project administration, Supervision, Writing – review & editing.
