# Supplementary material for: Myeloid-derived suppressor cells in influenza virus-induced asthma exacerbation
Source: Front Immunol. 2024 Apr 17;15:1342497. doi: 10.3389/fimmu.2024.1342497 (PMC11061804; doi:10.3389/fimmu.2024.1342497)
Supplement: Supplementary file 1 [file DataSheet_1.pdf]

Supplementary figure 1

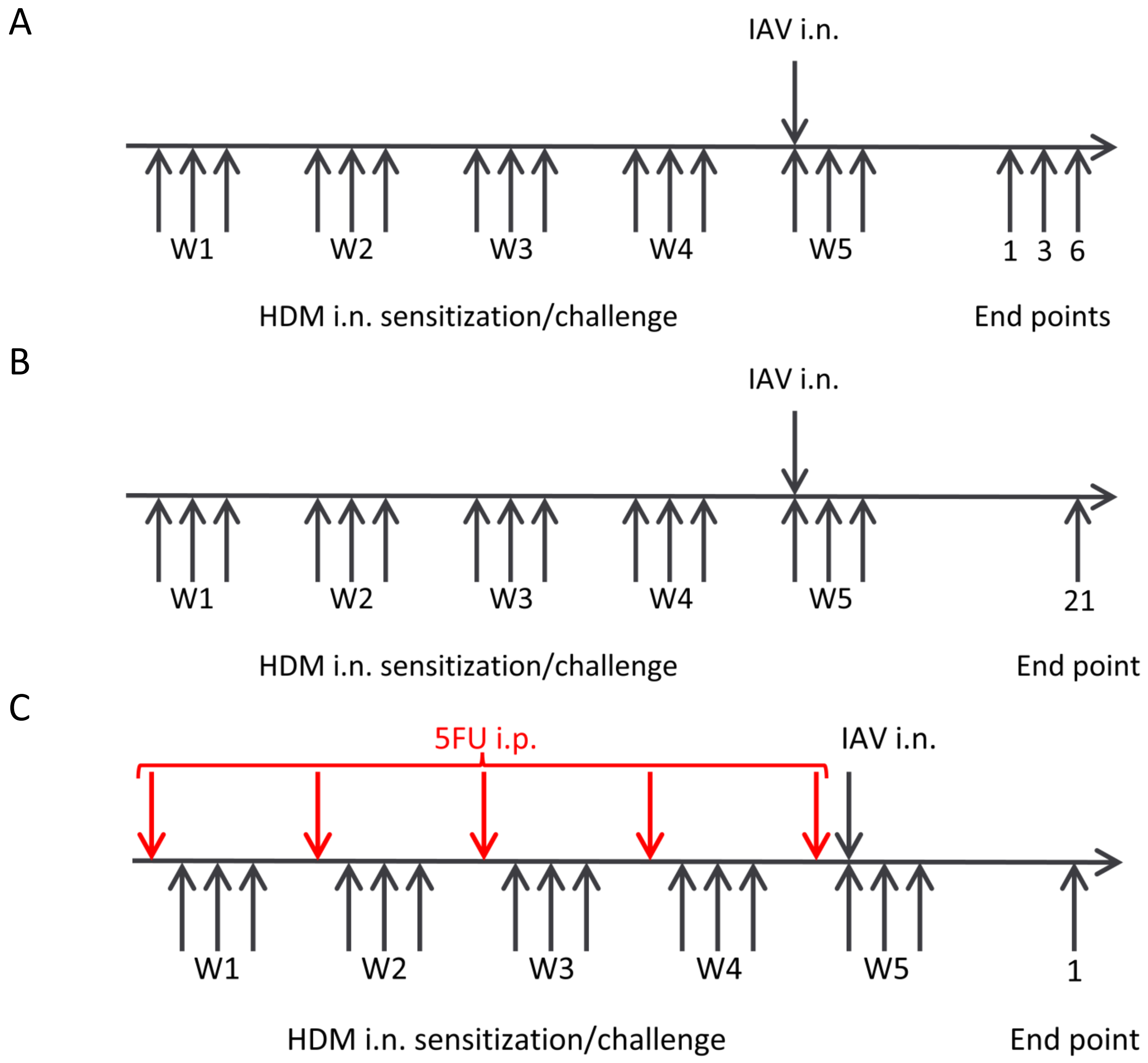

Supplementary figure 1 | Experimental timelines of the murine model of HDM- and subsequent IAV-induced asthma exacerbation. (A, B, C) Mice were i.n. exposed three times a week (W) for a total of five weeks to either PBS or HDM. (A) During the first HDM exposure of week 5 (W5), mice were additionally i.n. exposed to either PBS or $2x10^5$ PFU IAV. (A) One, three or six days after the final HDM exposure, mice were sacrificed for further analysis. (B) During the first HDM exposure of W5, mice were additionally i.n. exposed to $5x10^5$, $10x10^5$, $15x10^5$ or $20x10^5$ PFU IAV. (B) Mice were sacrificed 21 days post IAV infection for further analysis. (C) Mice were intraperitoneally injected with 5FU one day before the first HDM treatment of every week of the model, for a total of 5 doses. (C) One day after the final HDM exposure, mice were sacrificed for further analysis.

Supplementary figure 2

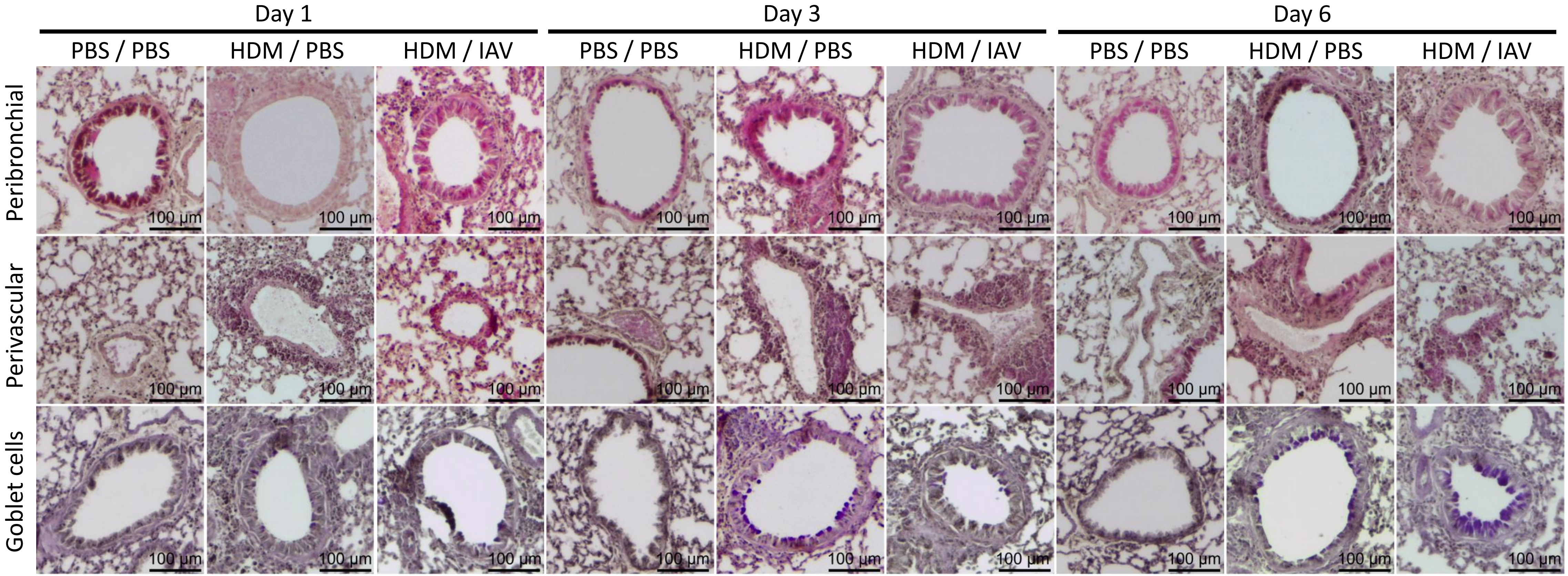

Supplementary figure 2 | Representative figures of lung inflammatory features induced by a murine model of asthma exacerbation. Mice were i.n. exposed three times a week for a total of five weeks to either PBS or HDM. During the first exposure of the fifth week, mice were additionally i.n. exposed to either PBS or $2x10^5$ PFU IAV. One, three or six days after the final HDM exposure, the lungs were collected and used to assess lung inflammatory features. Peribronchiolar and perivascular inflammatory scores were assessed by H&E staining, and the goblet cell score by PAS staining of lung sections. Representative microscopic pictures for the data presented in Figure 1A-C are shown (10X magnification).

Supplementary figure 3

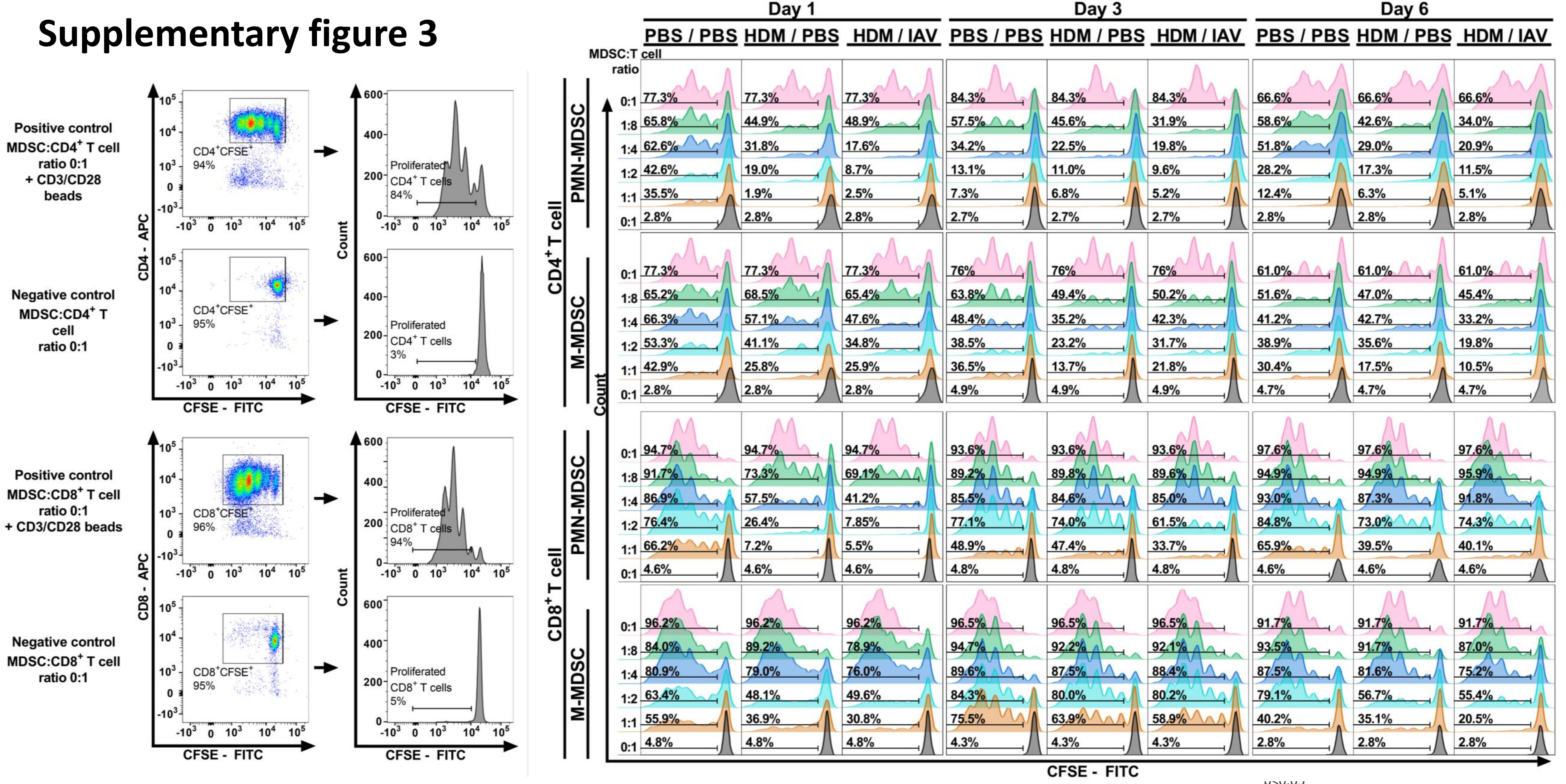

Supplementary figure 3 | Gating strategy and representative figures of the immunosuppressive activity of lung MDSCs in a murine model of asthma exacerbation. One, three or six days after the final HDM exposure, the lungs were collected. Both PMN- and M-MDSCs were isolated from the lungs by MACS separation. Isolated MDSCs were then co-cultured with CD3/CD28 biotin-beads-stimulated and CFSE-stained $CD4^+$ T cells or with $CD8^+$ T cells isolated from spleens of naïve donor mice at different ratios (MDSC:T cell ratios of 1:1, 1:2, 1:4 and 1:8). Proliferation of T cells was assessed after three days of co-culture by measuring CFSE dilution. The gating strategy as well as representative histograms of CFSE intensity are shown. Percentages of T cell proliferation were then normalized to positive controls, which were set to a 100% proliferation, and the results with normalized percentages are shown in Figure 4.

Supplementary figure 4

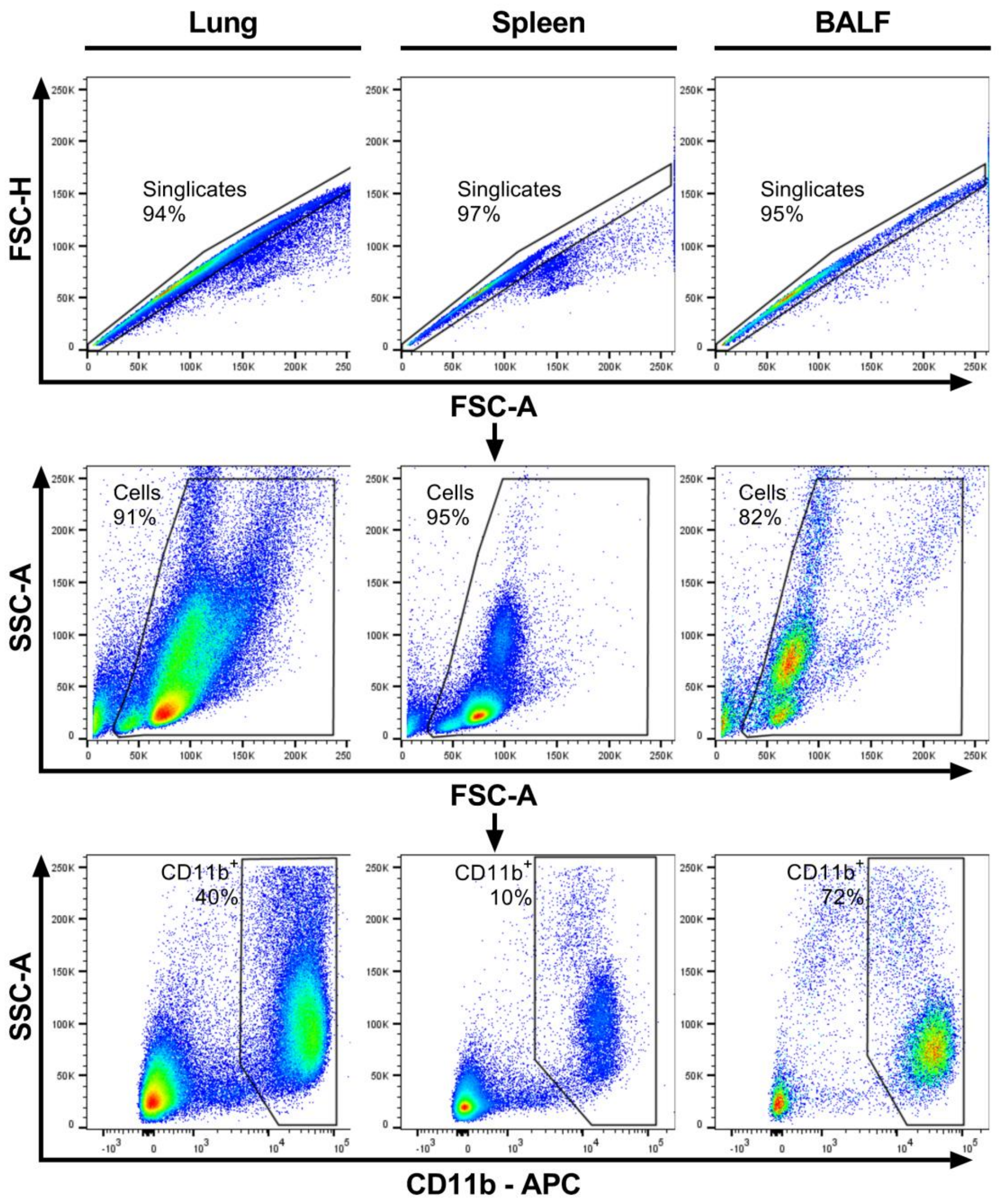

Supplementary figure 4 | Representative figures of the CD11b⁺ gating process of single cell suspensions of the lung, spleen and BALF preceding MDSC gating in a murine model of asthma exacerbation. Mice were i.n. exposed three times a week for a total of five weeks to PBS or HDM. One, three or six days after the final HDM exposure, the lungs, spleens and BALF were collected. PMN- and M-MDSCs were subtyped using flow cytometry, which was preceded by gating of viable CD11b⁺ cells. Representative figures of the gating process of viable CD11b⁺ cells preceding MDSC gating from the lungs, spleens and BALF are shown here. First, doublet cells were excluded by gating single cells in a forward scatter height (FSC-H) vs forward scatter area (FSC-A) density plot. Then debris was excluded in a sideward scatter area (SSC-A) vs FSC-A density plot. CD11b⁺ cells were then gated in an SSC-A vs APC density plot, with the APC fluorochrome being attached to a CD11b antibody. The gated CD11b⁺ cell populations were then gated for MDSCs based on the Ly6C and Ly6G markers as presented in Figure 3A and supplementary figure 5.

Supplementary figure 5

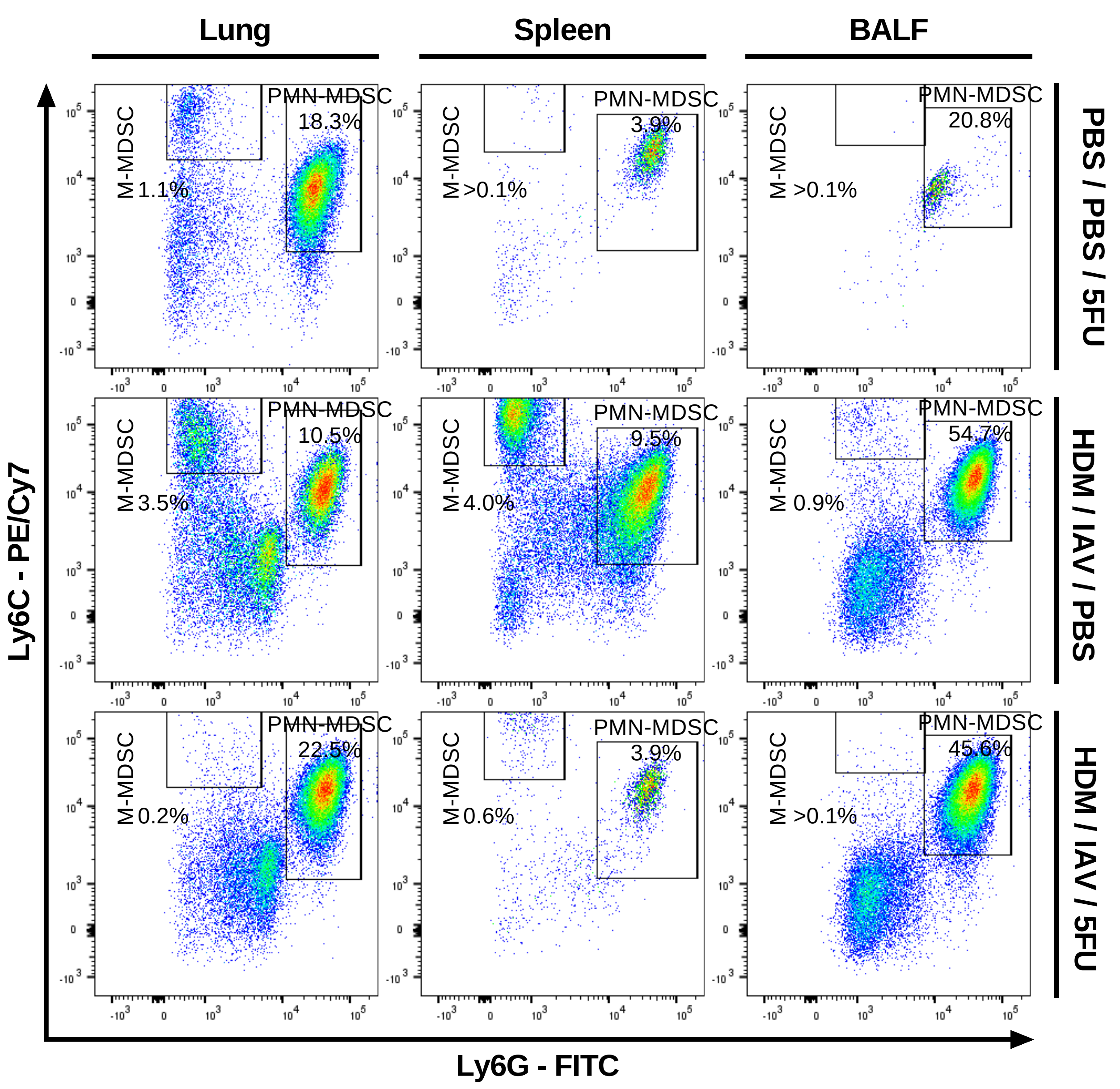

Supplementary figure 5 | Representative figures of MDSC gating process of single cell suspensions of the lung, spleen and BALF in a murine model of asthma exacerbation and 5-fluorouracil (5FU)-induced MDSC depletion experiments. Mice were i.n. exposed three times a week for a total of five weeks to either PBS or HDM. Starting one day before the first HDM challenge, 5-fluorouracil was administered i.p. weekly in an attempt to deplete MDSCs. During the first exposure of the fifth week, mice were additionally i.n. exposed to either PBS or $2x10^5$ PFU IAV. One day after the final HDM exposure, the lungs, spleens and BALF were collected. PMN- and M-MDSCs were subtyped using flow cytometry. Representative figures of the MDSC gating process of viable CD11b+ cells from the lungs, spleens and BALF based on the Ly6C and Ly6G markers as presented in Figure 5A are shown.

Supplementary figure 6

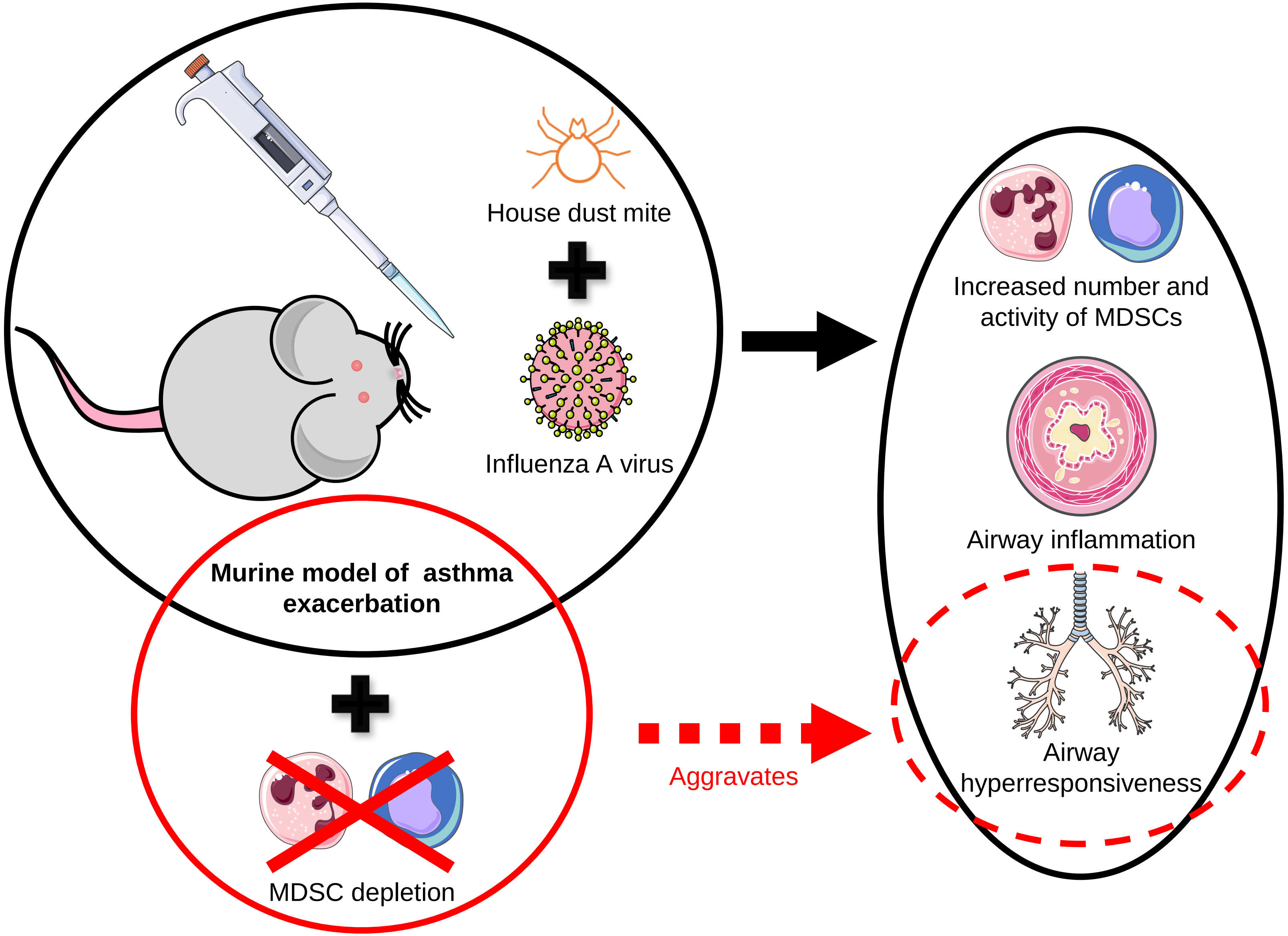

Supplementary figure 6 | Graphical representation summarizing the findings that provide some of the first evidence of the importance of myeloid-derived suppressor cells in a murine model of asthma exacerbation and their role in attenuating airway hyperresponsiveness. BALB/c mice were exposed to house dust mite three times a week for a total of five weeks to induce a chronic asthmatic phenotype, which was exacerbated by additional exposure to the A/Hamburg/5/2009 hemagglutinin 1 neuraminidase 1 (H1N1) influenza virus. Induction of lung inflammatory features and an increased airway hyper-responsiveness were observed, establishing the asthma exacerbation model. The number and activity of pulmonary MDSCs increased in exacerbated asthmatic mice compared to non-exacerbated asthmatic mice. Furthermore, depletion of MDSCs aggravated airway hyper-responsiveness in exacerbated asthmatic mice. The Figure was partly generated using Servier Medical Art, provided by Servier, licensed under a Creative Commons Attribution 3.0 unported license.
